# Feasibility and acceptability of a novel community-based mental health intervention delivered by community volunteers in Maharashtra, India: the Atmiyata programme

**DOI:** 10.1186/s12888-020-2466-z

**Published:** 2020-02-07

**Authors:** Kaustubh Joag, Laura Shields-Zeeman, Nandita Kapadia-Kundu, Rama Kawade, Madhumitha Balaji, Soumitra Pathare

**Affiliations:** 1 Centre for Mental Health Law and Policy, Indian Law Society, Pune, 411004 India; 2 Trimbos Institute (Netherlands Institute for Mental Health and Addiction), Da Costakade 45, 3521 VS Utrecht, the Netherlands; 3 Johns Hopkins Centre for Communication Programs, John Hopkins Bloomberg School of Public Health, Baltimore, MD 21202 USA; 4 Sangath, South Goa, Goa 403720 India

**Keywords:** Community mental health, Distress, Low and middle-income countries, Community-based intervention, Common mental disorders

## Abstract

**Background:**

Many community-based intervention models for mental health and wellbeing have undergone robust experimental evaluation; however, there are limited accounts of the implementation of these evidence-based interventions in practice. *Atmiyata* piloted the implementation of a community-led intervention to identify and understand the challenges of delivering such an intervention. The goal of the pilot evaluation is to identify factors important for larger-scale implementation across an entire district in India. This paper presents the results of a feasibility and acceptability study of the Atmiyata intervention piloted in Nashik district, Maharashtra, India between 2013 and 2015.

**Methods:**

A mixed methods approach was used to evaluate the Atmiyata intervention. First, a pre-post survey conducted with 215 cases identified with a GHQ cut-off 6 using a 3-month interval. Cases enrolled into the study in one randomly selected month (May–June 2015). Secondly, a quasi-experimental, pre-post design was used to conduct a population-based survey in the intervention and control areas. A randomly selected sample (panel) of 827 women and 843 men age between 18 to 65 years were interviewed to assess the impact of the Atmiyata intervention on common mental disorders. Finally, using qualitative methods, 16 Champions interviewed to understand an implementation processes, barriers and facilitators.

**Results:**

Of the 215 participants identified by the Champions as being distressed or having a common mental disorder (CMD), *n* = 202 (94.4%) had a GHQ score at either sub-threshold level for CMD or above at baseline. Champions accurately identified people with emotional distress and in need of psychological support. After a 6-session counselling provided by the Champions, the percentage of participants with a case-level GHQ score dropped from 63.8 to 36.8%. The second sub-intervention consisted of showing films on Champions’ mobile phones to raise community awareness regarding mental health. Films consisted of short scenario-based depictions of problems commonly experienced in villages (alcohol use and domestic violence). Champions facilitated access to social benefits for people with disability. Retention of Atmiyata Champions was high; 90.7% of the initial selected champions continued to work till the end of the project. Champions stated that they enjoyed their work and found it fulfilling to help others. This made them willing to work voluntarily, without pay. The semi-structured interviews with champions indicated that persons in the community experienced reduced symptoms and improved social, occupational and family functioning for problems such as depression, domestic violence, alcohol use, and severe mental illness.

**Conclusions:**

This study shows that community-led interventions using volunteers from rural neighbourhoods can serve as a locally feasible and acceptable approach to facilitating access social welfare benefits, as well as reducing distress and symptoms of depression and anxiety in a low and middle-income country context. The intervention draws upon social capital in a community to engage and empower community members to address mental health problems. A robust evaluation methodology is needed to test the efficacy of such a model when it is implemented at scale.

## Background

Progress has been made in improving access to mental health services for common mental health problems in many low- and middle-income countries (LMICs), but gaps remain in service provision and support options [1–3]. India’s National Mental Health Survey in 2016 estimated the mental health treatment gap between 83 and 85% [4]. Even after implementation of ambitious capacity building initiatives, India will not have sufficient numbers of mental health specialists to address the needs of persons with common mental health disorders (CMD) and mental distress. This is particularly the case in rural areas [5], as existing human resources for health are predominantly concentrated in urban settings [6, 7]. In addition, India’s public health system is already overstretched [8–11], and training existing health workers to take up additional tasks in mental health care may pose an arduous, and unsustainable task. Consequently, to complement services offered through the public health system, alternative approaches to identification, treatment and care for mental health problems are needed [5]. Models in recent years in LMICs have focused on task-shifting [12, 13], primarily through lay health workers providing counselling [14]. However, such service-delivery models also face challenges of acceptability, capacity building and financial sustainability. Increasing the capacity of existing informal community resources is one potential solution to increase the availability of mental health support and care in rural India for problems such as depression and anxiety. Many people that present with symptoms related to CMD may not require clinical care and could benefit from ‘talking-therapy’ based interventions, or interventions aimed at improving wellbeing and social support.

In many villages in India, there is a strong sense of community and willingness to help fellow members [15, 16]. Much of rural India works with an informal economy and employment is often seasonal or in clusters, particularly for work in the agricultural sector. There are many self-help groups (SHG) focused on discussing and carrying out income generation activities. These are democratically run, and their leaders are drawn from local communities. Examples of such groups include women’s income-generation groups and farmers clubs. There is a pool of village-level community members who have the time and inclination to provide support to other members in their community. The *Atmiyata* intervention was developed to tap into these community resources, or “Champions” [17]. It aims to build informal community care for people in distress and with symptoms of depression and anxiety, as well as facilitate access to social benefits, a core strategy for improving mental health and social outcomes for rural populations with poor access to formal mental health services [18, 19].

While community-based intervention models for mental health and wellbeing exist and have been subject to robust experimental evaluation, there are limited accounts of the implementation of these evidence-based interventions in routine practice. *Atmiyata* was designed to pilot the implementation of such a community-led intervention, to better identify and understand the challenges of delivering this intervention in practice, and with the ultimate goal of evaluating the intervention and then scaling this up to reach significant numbers in the community.

This paper reports the results of a feasibility and acceptability study of the *Atmiyata* intervention, which was piloted in Nashik district, Maharashtra, India, between 2013 and 2015.

## Methods

### Programme description

Atmiyata is a proof-of-concept study, funded by Grand Challenges Canada (Grant number: 0327–04). Details of the programme and its intervention components are described elsewhere [17]. Briefly, the *Atmiyata* intervention is an integrated care approach addressing both social and health care needs and consists of the following sub-interventions. The first is identification of community members with mental distress and CMD followed by provision of 6 basic counselling sessions by the *Atmiyata* Champion (Additional file 1). Counselling techniques include active listening, behavioural activation, problem solving, and social support. These components were selected on the basis of empirical evidence of effective strategies for people with CMDs [20–22].

Along with this, Champions were trained in qualities of a good counsellor, giving support and reassurance, using relaxation technique. For first session which is called as Session 0, Champions identify if person is going through distress or having CMD and willing to take 4 to 6 sessions of counselling. During the sessions, Champion use active listening throughout all sessions, and use activity scheduling for issues of inactivity, lethargy, not going to work, lack of interest in doing anything and problem-solving techniques to better understand a problem and make appropriate decisions. Relaxation technique was used during 3rd to 6th session and as needed. Each session had home-work assignments for activities or problem solving. Champions were also trained to summarize each session with the beneficiary. At the end of 6 sessions, take-away points were discussed. If beneficiary needed more than 6 sessions, then she or he was referred to a mental health professional (Additional file 2).

A structured training manual for Champions and Mitras in English & Marathi was developed as part of the intervention. Champions received a 7-day training delivered over 3 weeks from the project lead and project manager on behavioral activation including theory of behavior and its connection to emotions, cycle of inactivity and activity; activity scheduling and motivating a person to take on small tasks and monitor and follow-up. Champions also received a 1-day refresher training every 3 months. Project lead and project manager supported and mentored community facilitators (CFs) and CFs in turn supervised and mentored Champions. CFs met Champions twice a month. Project lead met with CFs and project manager once in a month to oversee the implementation and provide mentoring and supervision. CFs during their monitoring visits asked Champions about counselling sessions and with consent, sometimes met the beneficiary to understand the process of counselling. These supervision sessions ensured fidelity to the intervention delivery.

*Atmiyata* Mitras are a separate but complementary group of community volunteers receiving comparatively less training, whose main duties are to identify distress among village members and refer them to the Champions for further support, as well as provide information about distress and wellbeing to community members (Additional file 1). The second sub-intervention consists of ‘narrow-casting’ films to raise community awareness regarding mental health. These are loaded on mobile phones, are short scenario-based depictions of problems commonly experienced in villages (such as alcohol use or domestic violence) and are designed to be a source of inspiration for solutions which community members themselves can develop and employ in their own communities. Third, Champions facilitate the process of applying and securing social benefits for persons with CMD and severe mental disorders (SMD). These are defined as government (either national level or state level) allowances or (financial) benefits granted to certain vulnerable populations, such as women or unemployed individuals. The Champions help identify community members who are eligible for these allowances and assist them in the application process. Finally, referrals are made by Champions to specialised health services, based on the nature and severity of symptoms. Community members at risk for suicide or with primary complaints of headaches or insomnia are referred to the nearest primary health centre (PHC). If the PHC doctors are unable to treat these problems, referrals are made by the PHC doctors to a psychiatrist based at the public-sector district hospital (referred to as “Civil hospital” by Champions). Persons with a potential SMD (severe depression or psychosis) are referred directly to the psychiatrist at the district hospital. Follow ups are subsequently done over the telephone or in person by the Champion.

### Setting

The *Atmiyata* intervention was implemented in the Peth block [sub-district] which is about 50 km from the city of Nashik, the capital city of Nashik district in the Indian state of Maharashtra. The district of Nashik has a population of approximately 6.1 million, with 3.5 million people living in urban areas and 2.5 million people living in rural areas. It is divided into 15 blocks (or sub-divisions). Trymbak, the neighbouring block to the intervention block, was chosen as the control block, as it had similar socio-demographic characteristic as Peth block. The Trymbak block (control) has 125 villages in total with population of 156,367; the Peth block (intervention) has 145 villages with population of 119,838 [23]. The total adult population covered in the intervention area was 14,000 across 40 villages.

Both the intervention and control blocks are economically deprived, have poor transportation connections and limited access to health care. Scheduled tribes, a group of people who fall “outside” the caste system (and form the lowest rung in India’s social hierarchy) constitute more than 90% of the population in these blocks [23, 24]. The sample is characterized by low literacy, with only 20% having attended high school. About half the sample is less than 30 years old, 30% are in the 30–39 age group and another 20% are between 40 to 65 years. Majority of the respondents lived in households that are made of mixed materials like mud and stone and only 5% live in brick homes. Large families (5 to 8 members) seem to be a norm with only 23% respondent living in families with four or less members.

In terms of social groupings about 97% of the respondents belong to Hindu tribal groups and 3% comprise of Muslims and Buddhists. The two major tribal groups in the area are the Konknar (46%) and the Hindu Mahadev Kolis (36%).

### Study design

This was a feasibility study that employed a mixed-methods approach (both quantitative and qualitative methods) to understand to if the *Atmiyata* programme was feasible to implement, acceptable to beneficiaries and to implementers, and appropriate for the local cultural context.

#### Quantitative methods

Two quantitative approaches methods were used for evaluation. The first approach focused on measuring impact of the intervention on a subset of CMD cases identified by the Champions. A month of implementation (May and June 2015) was randomly selected and cases detected during this month were enrolled into the study. The aim was to assess the ability of Champions to accurately detect CMD, and to examine the impact of the intervention on levels of distress. The second approach used a quasi-experimental intervention control design using a panel at baseline and end line.

#### An assessment of CMD cases

A pre-post survey was conducted with 215 cases identified with a GHQ cut-off of 6 using a 3-month interval. Cases were enrolled into the study in one randomly selected month (May–June 2015). These cases were administered the General Health Questionnaire (GHQ-12) both before and after 3 months of receiving counseling from a Champion. The Atmiyata counsellors saw 1150 cases during the one-year intervention.

#### Population-level survey

A panel survey was conducted in both intervention and control blocks with women and men at baseline and at 12-month follow-up to look at population level changes in GHQ-scores. The same individuals were interviewed at baseline and end-line. Peth block was selected for the intervention and a neighbouring block, Trymbak was chosen as the control area because of similar geographical and socio-demographic characteristics as Peth.

Sample size was calculated with the aim to increase treatment seeking for mental illness from 10 to 20%. With a power of 90%, and *p* value of 0.01, the sample size was 397 women and 397 men [25]. Another 15% was added to account for attrition and clustering. The estimated sample was approximately 900 participants per block i.e. 450 women and 450 men per block (*N* = 1800). The final baseline sample included a total of 1857 respondents who were administered the survey. At end line, the final sample was 1670, indicating an overall dropout rate of 10%. The dropouts occurred primarily due to respondents’ travel during end line data collection. The data presented in the paper includes a panel of 829 respondents in the intervention area and 841 respondents in the control area.

Twenty villages were randomly selected in each block. For the selected 40 villages, a systematic random sample of women and men was generated using a random start from the sampling frame that was obtained from the 2014 online electoral rolls of Maharashtra.

We used a stratified systematic random sampling technique which enables random sampling at each strata of the population for example, village, women and men. Stratified sampling results in a more representative sample.

The baseline survey was administered between August and September 2014 and the end line survey was administered in August and September 2015. The surveys were administered by interviewers employed by the project, all of whom were graduates in social sciences. Interviewers participated in a training program led by the principal co-investigator (NKK) on how to administer the survey. Informed consent was obtained from each participant, and the survey was administered on paper. The survey took approximately 60 min to complete.

#### Outcome measures

The outcome measure for both the population-level survey and the CMD case assessment was the 12-item General Health Questionnaire (GHQ-12) [26]. The GHQ-12 has good psychometric properties and is one of the most rigorously validated screening tools for CMDs in India, with Cronbach’s alpha of the one dimension GHQ model and test-retest reliability scores consistently above 0.8 [27–29]. In our study, Cronbach’s alpha for the GHQ 12 was 0.81.A GHQ cut-off score of 6 was applied, which is consistent with previous studies in India [14, 30].

#### Data analysis

For analysis of the CMD sample drawn, as the aim was to assess whether there was a higher proportion of cases above the cut-off for CMD at 3 months after the counselling sessions, a chi-square test for within-subjects designs to test for analysis of binary dependent variables was applied using the McNemar-Bowker test of symmetry.

For the population-based survey the analysis included examining non-cases (GHQ 0–3), sub threshold cases (GHQ 4–5) and cases (GHQ 6+). A logistic regression gain score analysis was used to assess the relative changes in GHQ-12 scores over time. The difference in GHQ scores was calculated as: GHQ score at end line-GHQ score at baseline. A binary variable was generated as 0 for “positive gain” and 1 for “negative gain”. Negative gains were expected after the intervention. The differences in scores were entered as the dependent variable and the block (0 = Tryambak (control) and 1 = Peth (intervention block)) was the independent variable. The data analysis was conducted using STATA version 13.

#### Qualitative methods

An important part of assessing the acceptability and feasibility of *Atmiyata* was to ascertain what it was like for Champions to be working in their communities in their new role. In-depth interviews were conducted with 16 Champions to explore factors which motivated them; their experiences of delivering the intervention, including their ability to detect affected persons; problems or difficulties faced in this process; and finally, benefits the community received. Unfortunately, we were not able to carry out interviews with intervention and control participants due to logistical challenges, particularly in research team capacity.

Interviews were coordinated by the co-principal investigator (NKK) of the project team and carried out by a trained researcher in the field. Interviews were conducted in Marathi in March 2016, and audio recorded. They were transcribed directly into English using Microsoft Office by a native Marathi speaking researcher who was fluent in English.

Interviews were analyzed using thematic analysis, a process involving *“identifying, analyzing and reporting patterns (themes) within data”* [31]*.* Analysis was conducted in “Dedoose”, a web-based software for qualitative methods. Codes were developed and the coded excerpts were extracted from Dedoose. The final analysis is structured by the main themes which emerged across 16 interviews.

### Ethical considerations

Ethical approval was obtained from the institutional review board of the Indian Law Society (approval Number-ILS/77/2014). All participants surveyed or interviewed provided the written informed consent to complete the surveys. Names of participants and other identifiers did not appear on the questionnaires and de-identified data were analysed. Research assistants conducting the surveys were trained on the consenting procedure.

## Results

### Socio-demographic profile of the sample

Champions identified 215 persons with distress in May–June 2015. Of these, 48% were women and 52% were men. About 29% of the cases were non-literate, 18% an education from 1 to 4th grade and the majority (43%) had studied between 5 to 10th grade. Only 10% of the cases were educated beyond 11th grade.

The panel included 829 respondents in the intervention block and 841 respondents in the control block. The sample had approximately equal numbers of men and women. Over 75% of the sample were between the ages of 18 and 39. The majority worked as labourers on farms and lived in a joint family with 5–7 members. The only statistically significant difference in demographics between the two blocks was that the control participants had significantly lower educational levels (Table 1).

**Table 1:** Socio-demographic characteristics of the population study participants at baseline from intervention and control group

Characteristics		Intervention (*n* = 829)	Control (*n* = 841)	*p* value
Gender	Female	49.9	49.2	0.583^ns^
	Male	50.1	50.8	
Age (years)	18–29	45.7	52.7	0.108^ns^
	30–39	31.0	29.6	
	40+	23.2	17.6	
Education	No school	26.7	39.0	0.000^a^
	Up to 4 years of schooling	13.1	14.9	
	5–10 years of schooling	35.3	34.2	
	11+ years of schooling	24.8	11.9	
Occupation	Farmer	72.1	79.3	0.201^ns^
	Labourer & worker	23.0	15.9	
	Not working	4.8	4.7	
Family type	Joint	82.6	79.9	0.279^ns^
	Nuclear	17.3	20.1	
Number of members in family	1–4	26.5	19.6	0.088^ns^
	5–7	48.7	54.7	
	8+	24.7	25.6	
Type of house	Kutcha	24.2	24.5	0.534^ns^
	Kutcha-Pucca	71.5	69.6	
	Pucca	4.2	5.9	
Place of defecation	Toilet	27.4	28.3	0.439^ns^
	Open defecation	72.6	71.7	

*Values are percent of the participants*

*Test of proportion* i.e. *Chi-square test was used to test the difference in proportion across the intervention and control group*

*ns Statistically non-significant difference in proportions (p > 0.05)*

^a^*Statistically significant difference in proportions (p < 0.05)*

Of the 59 Champions, 16 (27%) participated in the in-depth interviews, of which 8 participants were male and 8 were female. The age range was between 28 and 45 years. The average Champion had completed at least primary school.

### Case identification skills of Atmiyata champions

Of the 215 participants identified by the Champions as being distressed or having a CMD, 202 (94.0%) had a GHQ score at either sub-threshold level for CMD or above at baseline. This indicates that the Champions were able to accurately identify both people who had emotional distress as well as those with significant problems in need of psychological help and assistance (Table 2). The qualitative interviews support this finding:

*If I have to identify a person with stress or tension, that person would have negative thoughts, must be in his own world, would not talk to anyone, be silent etc. Irritability, exhaustion, not showing any interest in any work … (these) are the symptoms (*Champ60222M01)

Champions were trained to follow a specific referral protocol when support needed exceeded their responsibilities and capabilities, or when they identified potential severe cases of distress and mental ill-health. Champions referred 77 cases of CMD and 181 cases of SMD tospecialised mental health services in one year (Table 3).

*My friend (MD) … in frustration MD started drinking so much that in the morning instead of drinking water MD used to drink alcohol. MD’s body used to jitter. MD used to get angry seeing those films. When I realized that MD was beyond my help I took MD to Civil (hospital). (*Champ60224M04)

**Table 2:** GHQ 12 scores for persons with distress (*n* = 215) at baseline and 3 Month follow-up

GHQ12 Scores of persons with distress identified by Champions	May–June 2015 Persons with distress detected by *Atmiyata* Champions (*N* = 215)		3 month Follow-up of persons with distress (*N* = 215)	
	N	%	N	%
Non-Case (0–3)	13	6.0	85	39.5
Sub Threshold case (4–5)	63	29.3	52	24.2
Case (6+)	139	64.7	78	36.3
Total	215	100	215	100

*The McNemar-Bowker test determined the significant difference in the proportion of cases before counselling and at follow-up (p < 0.001)*

**Table 3:** Overview of *Atmiyata* Champions, Mitras and service utilisation rates

Variable	*N* (%)
Number of Champions selected and trained	
Total	65
Male	37 (57%)
Female	28 (43%)
Retention rate of Champions until the end of implementation	
Total	59 (91%)
Male	33 (56%)
Female	26 (44%)
Number of *Atmiyata* Mitras selected and trained	264
Retention rate of *Atmiyata* Mitras until the end of implementation	264
Identification and service utilisation	
Number of people with common mental health problems detected	1150 (8.2% of population)
Number of people with common mental disorders referred to primary and secondary health services	77
Number of people with severe mental health problems detected	181 (1.3% of population)
Number of referrals of severe mental health problems to specialized care	181 (1.3% of population)
Number of people who used referrals	92 went to public health facilities (51% of those referred) 28 chose to go to private providers (15.5% of those referred)
Number of people with severe mental health problems (in public health facilities) that continued treatment after referral	58/92 (63% of those who went to public facilities)
Number of families assisted to obtain social benefits like Sanjay Gandhi Niradhar Scheme, Integrated Watershed Management Program, Life Insurance for people with disability, Mahatma Gandhi Rural Employment Scheme.	276 (1785 individuals in families12.75% of population)
Direct monetary benefits received by	624 individuals
Treatment seeking for persons with CMD- % of respondents seen by Atmiyata Champions at end line population survey	17.5%

### Acceptability and feasibility of the intervention and implementation

#### Acceptability and feasibility of the training

Atmiyata selected and trained 65 volunteers (37 males and 28 females) as Champions. Of these, 8 dropped out after training and were replaced with 2 new Champions. Thus, a total of 59 (90.7% of the initial selected champions) continued to work until the end of the project (Table 3).

After being selected, some Champions were concerned if they could perform their new community roles well; if they could use their mobile phones in a meaningful way to engage communities, share information about mental health to community members, and deliver counselling sessions. The training they underwent covered these topics and alleviated these concerns.

*We got a number of trainings from the programme people. They gave us courage; our fear is gone. We really received good exposure.* (Champ60224M11)

#### Acceptability and feasibility of intervention components

##### Counselling

Champions were able to detect and provide counselling sessions to 1150 people in distress (Table 3). Champions trained to deliver 4 to 6 sessions, depending upon need and as agreed between the Champion and the beneficiary to deliver behavioral activation, problem solving and training in relaxation exercises. The details of 6 sessions is attached as supplementary material. Champions were trained on symptoms of distress and mental health issues and to distinguish between common and severe mental disorders. Champions identified people with CMD through symptoms. About 17.5% percent of the respondents in the population survey self-reported that they received the intervention from the counsellors (Table 3). This self-report may be an under-estimate due to recall issues and counselling sessions happening in informal settings, so beneficiaries not perceiving them as counselling sessions. The qualitative interviews highlighted the adverse consequences of these sessions; that could occur if the problematic behavior or thought pattern continued; and included suggestions of how to change these.

*One-person (AB) … AB got married but the partner (left AB and) went back (to own) home the next day … AB’s partner refused to come back … AB was in great tension. I told AB, “Don’t think so much. If you think so much then it is you who will be stressed. When people start thinking they think big (magnify problems)”. I told AB, “You have a brother, a sister in law, take their support. Put your mind on your work.” … soon AB was better. Now AB says that AB will marry again.* (Champ60224M03)

Champions also counselled families of affected individuals on how they could change their own behaviors and help one another.

Champ60222M02*: A patient’s (DB) partner was staying at parent’s house, and later married DB’s younger sibling. Since then DB’s partner had stopped paying attention to DB. It had affected DB’s mental health and DB was suffering from depression. If asked, DB would complain of headaches. I spoke to DB’s partner, DB’s mother and children. I told DB’s children to give special attention to DB and to take extra care, and make DB feel wanted, I said “This will help your parent to get well soon and back to everyday routine”. Earlier DB had stopped taking care of self, had stopped combing hair, taking a bath. But now DB is fine and back to normal.*

##### Films

Monitoring data indicates that 7622 people (54.4% of the intervention population) viewed the films loaded on smart phones provided to the Champions. Atmiyata films were shared on the phones of 336 other community members through offline sharing technologies such as Bluetooth. Champions also screened the films at self-help group meetings or other community group meetings; informal and seasonal gatherings; and individual households. These films were very popular, especially among young people. Many villagers had experienced the problems depicted in these films and could identify with and understand the content.

*(I showed a) .. film in which a wife receives a phone call from her male friend and her husband listens to the call standing behind. Then he suspects her of talking to someone and beats her … such films were liked by the people. (*Champ60224M04*)*

*The films on mother in law and daughter in law (relationships) were liked by many women. Women liked the film on addiction. They (have) experienced it themselves. (*Champ60222M01)

##### Social benefits

276 families were assisted by the Champions to apply for social benefits in the state of Maharashtra. Social benefits included government schemes for vulnerable and marginalized sub-populations. For example, Champions facilitated following social benefits: Sanjay Gandhi Niradhar Scheme, Integrated Watershed Management Program, Life Insurance for people with disability, Mahatma Gandhi Rural Employment Scheme (Table 3).

*I had told one widow … She was in tension, (she said) “How to manage two daughters and one son?” Then I informed her “There is a widow pension scheme and you can apply”. (And) I told a person. he is now handicapped. I told him to gather all the documents like his Aadhar card (Government identity number), (told him) “Get yourself a handicap certificate and then you will get some money”. He submitted (the documents).* (Champ60224M03)

#### Acceptability of champions

Champions were well known in the community, and their strong presence facilitated their work. People felt comfortable talking to them, trusted them and appreciated their work.

*Everybody has respect for me in the village … after (showing) the films, taking patients, people recognize me even better. When they (people who received Atmiyata) felt better … they told others about how they went to the hospital, got the medicines and are now feeling better etc., then the other person would say “Even I can’t sleep, I also experience numbness”. They used to come and say to me, “Do something for me also.” (Then) I spoke to them. (*Champ60224M01)

*(A patient’s wife) She says “Because of you, my life has become very happy. My husband used to sit on the cot and was always in tension, would forget about his work but from the day you spoke, he is very free.” I feel happy to hear this. (*Champ60224M03*)*

Champions enjoyed their work and found it fulfilling to help others. This made them willing to work voluntarily, without pay.

*People used to ask us “Are you doing this job because of payment? “I explained to them that we don’t get any payment. We have to do social work, volunteer ourselves for good work. The aim is how people can live happily, healthier mentally and physically as well. One person asked, “If you don’t have any profit why do you work here?” I told (that person) “I like it, that is why I do (it)”.* (Champ60223M03)

*I feel good when I do something for someone. Because of me if someone’s life improves, I feel happy.* (Champ60224M11)

Some champions were cautious in how they approached people and attempted to earn the trust of others before helping them, often employing creative tactics. They were sensitive to the needs of others, and respected community norms.

*We started working in the village. But since the topic is sensitive, we did not tell the village that we were here for this particular project. During village visits, based on the symptoms told to us, we started identifying people with mental stress problems*. (Champ60223M03)

*In the beginning I did not show the films directly. I first won their heart and made a place in their heart. I made them understand what qualities I have and only then I showed them the film. (*Champ60224M04)

*We used normal words and explained things while chatting. We did not make them (people) feel like they were mental, and we were treating them … If anyone from the patient’s family asked me why I had come, I used to tell them that I had come just to chat. While chatting, we used to get to the topic, and I would make them talk about the problem and try to explain to them.* (Champ60222M02)

#### The Mitras’ work

To complement the work of the Champions, 264 community members were trained to become *Mitras* (Table 3). *Mitras* provided an understanding about mental health and *Atmiyata* to others in the villages, and helped identify cases, and referred them to the Champions. Mitras helped Champions to reach out in different areas in villages especially to people living on the outskirts of the villages. Mitras also helped address gender sensitivities, for example, if there was a male Champion in a village, he was assisted by 2–3 female mitras and vice-versa. Mitras were also helpful in addressing caste barriers (which are common in Indian villages) and thus help us reach all castes in the population.

*They (Mitras) shared information with the villagers. They showed films to the villagers. We worked together towards our aim.* (Champ60223M04)

#### Impact and benefits of the intervention

Table 2 describes the impact of the Atmiyata intervention on 215 persons identified with distress by Champions in May–June 2015. About 65% of the cases were accurately detected by the Champions as persons with CMD as they had a GHQ score of 6+. Another 29% were sub threshold cases (GHQ 4/5) and 6% were non-cases (GHQ 0–3). All the persons with distress received the counselling sessions from the Champions. At 3 months follow up, persons with GHQ scores 6+ (CMDs) had dropped from 65 to 36% (Table 2).

The other evidence of impact comes from the population-based pre-post survey data. The mean GHQ 12 score in the intervention group at baseline was 2.68. It reduced to 2.51 after one year of the intervention indicting a significant improvement as measured by a t-test (Table 4). Similarly, the control had a baseline mean GHQ 12 score of 1.50 which increased to 2.19 after one year. Data indicate a 43% global reduction on GHQ-scores from baseline to end line in the intervention group compared to only a 29% reduction in the control group (Table 4). The population-based study shows that the intervention group performed significantly better than the control group in terms of the primary outcome, reduction in CMDs (Table 4).

**Table 4:** Population-level impact on reduction in mean GHQ scores in the intervention and control areas

GHQ12 score	Intervention *N* = 829	Control *N* = 841
Baseline (Aug-Sept 2014)	2.68 ± 0.08	1.50 ± 0.05
End line (Aug-Sept 2015)	2.51 ± 0.07	2.19 ± 0.06
Change in mean GHQ score from baseline to end line	− 0.16 ± 0.10^a^	0.69 ± 0.08^b^
Percent of respondents with decrease in scores	43.1	29.2^c^

*Values are Mean ± Standard Mean Error*

*a. Between groups change over baseline was assessed by independent sample t test*

*The scores were significantly reduced in Intervention group as compared to control (p = 0.046)*

*b. Change over baseline was significant as assessed by paired t test (p = 0.001)*

*c. DifferenceinproportionwasstatisticallysignificantasassessedbyChi-squaretest (p = 0.001)*

A logistic regression analysis was carried out to assess the change in GHQ scores in intervention and control group after controlling for baseline scores. The difference in scores entered as dependent variable and group (0 = control and 1 = intervention) as independent variable. The analysis indicates that the intervention group has 1.3 (95% CI-1.1, 1.6) (R^2^–0.17) times more chance of a reduction in their GHQ score compared to control group (*p* < 0.05) (Table 5).

**Table 5:** Logistic regression for assessment of impact of intervention on GHQ score

Independent variable	OR	95% CI for OR	*P* value
Control area	1		
Intervention area	1.3		
		1.1–1.6	0.023*
GHQ at base line	0.5	0.4–0.6	0.000*

Dependent variable: Decrease in GHQ score (Yes/No)

Loglikelihood = − 953.26287

PseudoR^2^ = 0.1715

The qualitative in-depth interviews indicated that persons in the community experienced reduced symptoms and improved social, occupational and family functioning for a variety of problems such as depression, domestic violence, alcohol use, and severe mental illness. Employment, and family functioning also improved. These were attributed to a combination of intervention components. (Additional file 3)

*In one family, the husband used to beat his wife, when I came to know about them, I visited them. I counselled them. Showed them the films on quarrels, addiction. Now fights between them have reduced to a large extent. (*Champ60223M05)

Champions also reported positive changes in their own lives.

*I used to be very irritated. But now it is over. (Now) I take a deep breath. (*Champ60224M03)

*I have prevented many widows and separated women from suicidal thoughts. I myself had such thoughts but I overcame them. (I would think) “How can I do such things? I am the Champion of the project!... people will say “She used to counsel us and now she herself had tried this!”. These thoughts changed my mind-set. Now (I) am fearless and I feel confident.* (Champ60224M11)

#### Challenges

Some community members had low awareness about mental health problems or expressed scepticism regarding the Champions’ work and their motives and resisted the intervention.

*(I) took him (a patient) to Civil, got medication for him. But he did not take the medicines … he was the same … in his trance. People in our area are mainly superstitious. They don’t have much faith in medication. We all (project staff) tried our best. His family members were not convinced. The vehicle was available, still they were not ready (to take him again to Civil). (*Champ60224M10)

*She (a person) has left her husband and is staying alone, so I spoke to her brother about it and he told me, “What do have you to do with this (matter)?. She will look after herself!”. I tried telling the parents, but nothing happened. (*Champ60224M01)

Some families of Champions objected to them working in Atmiyata as it was time consuming and there was no remuneration given for efforts. However, many changed their minds once they came to know about the importance of the work. Family members frequently helped the Champions, for example, in approaching persons in the community, or handling the mobile phone. Champions complained of the long and laborious application process and the bureaucracy involved in getting access to social benefits.

*For just one paper I had to go many times. Every time it’s not possible for everyone. Money, time … all goes waste. (*Champ60222M06)

*There is only one lady (whose husband has left her). She is young still. I sent her to the Talalthi to get information on the various programs. I also gave her in writing the documents needed. The Talathi asked for the death certificate of her husband. But he is not dead. She is a destitute … but the Talathi is not listening. (*Champ60224M04)

Some Champions were also not able to help those with very severe symptoms of mental health problems. Other barriers included minor problems with using films (the voice quality was low, the screens of the phones were too small for proper display, or there were difficulties transferring films to other mobiles); the local hospital not having the necessary medication, the hospital being far away, which affected follow up; and some Mitras not having time to carry out their responsibilities.

There are challenges related to Implementation and health systems. Implementation related challenges included the following: 1. Champions found it difficult to attend training, which was addressed by arranging training venue, which was centrally located, arranging transport to training venue and planning dates by consensus. 2. Champions motivation was a challenge and we used few mitigation strategies as discussed above. 3 Champions had some difficulty using the app and smartphone which was addressed by ensuring that CFs provided continued support and during refresher sessions.

Health system related challenges included the following: 1. The Primary Health Care Centre had psycho tropic medicines but the doctors there felt they were not sufficiently trained and hence unwilling to prescribe them. 2. Similarly, the district hospital was willing to provide treatment, but had administrative difficulties in providing disability certification and thus none of our project beneficiaries obtained a disability certificate despite repeated efforts of the project team to address these barriers.

## Discussion

This study assessed the feasibility of a low-cost community volunteer led intervention to address mental distress, common mental disorders, and severe mental disorders.

There are several notable findings from this study. First, the model of capacity building employed to equip Champions with skills in identification, counselling and follow-up resulted in identification of CMD with reasonable accuracy. Admittedly, our data was only able to assess specificity of assessment and not sensitivity. To get an understanding of sensitivity, the National Mental Health Survey in India estimates the prevalence rate of CMD to be 6.3% [4]. Comparatively, the Atmiyata baseline community survey prevalence rate (GHQ case score 6 or more) was 14.2% (9% at end line in the intervention block compared to 2.2.% at baseline and 6.7.% at end line in the control block, respectively) while the Champions had a case detection rate of 8.2% (1150 out of 14,000).

Second, there was a difference in the number of cases (on GHQ-12 scores) becoming sub-threshold cases or non-cases before and after the 6 sessions of counselling provided. This may point towards a trend in the intervention contributing to a reduction in the severity of depressive and anxiety symptoms, as well as potentially preventing sub-threshold symptoms from exacerbating into a GHQ-score (thereby becoming a ‘case’), and thus warranting treatment. These findings complement the findings of other studies in LMICs that employed an intervention designed to reduce CMD, such as the Friendship Bench programme in Zimbabwe [32] and the MANAS programme in India [14]. The central finding across this body of research is that task-shifting to other cadres of health workers and, in our case, community volunteers can reduce symptoms of depression and anxiety, and can contribute to improved wellbeing and less distress. These community-based local models of mental health care support are context-driven and culture specific. Across programmes like the Friendship Bench and *Amtiyata*, the core idea of the intervention remains the same, but tailoring of the intervention and subsequent implementation takes different shape in different contexts, to reflect local realities. In our experience, accessibility and acceptability of the intervention was strengthened as locally accepted and understood terms of distress and wellbeing were used, moving away from medicalised terms such as depression or anxiety. Indeed, access to and acceptance of treatments can be enhanced when local understanding are used instead of more clinical and biomedical conceptualisations [33].

**Fig. 1:**
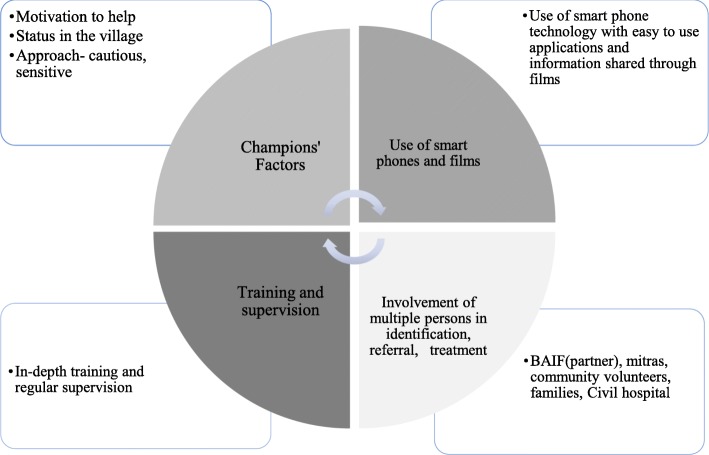
‘Components responsible for *Atmiyata’s* success’

Third, both qualitative and quantitative findings reveal that the intervention was feasible for implementation in a rural setting in India. It was acceptable for recipients to receive support from a known person in the community, and acceptable for the Champions to deliver these supports. The leadership roles they played in the community, their manner of approach and their intrinsic motivation to support their communities are important factors. Scaling-up community volunteer-led models must be carefully thought through; the debate is ongoing as to whether it is ethical to not incentivize volunteers, particularly in disadvantaged or impoverished communities [34]. Some programs have provided incentives in the form of bicycles, mobile phones, and clothing, while others offer small stipends or sitting allowances to conduct activities [35]. A balance needs to be achieved between upholding the spirit of volunteerism in the health care sector with economic needs. The programme was able to retain the majority of the trained Champions throughout the course of programme implementation. There may be several reasons behind the high retention rate and sustained commitment to delivery of the intervention. Firstly, the project team carefully selected Champions (focusing on community leaders, for example), and employed engagement strategies for keeping up their enthusiasm level to continue working. Secondly, the supervisors (2 part-time supervisors per 60 champions) [17] played a key role in sustaining the motivation of Champions, as did the supervision system, which focused on providing hands-on support and coaching to champions within their own real-world working environment. Thirdly, monthly meetings brought Champions together to share experiences or insights and was fruitful in maintaining group cohesion, and in providing a peer-to-peer platform for enhancing ways of working or skills used in the field. Finally, selecting Mitras that were willing to support the work of the Champions enhanced the Champions’ ownership over the process of providing community support. There were also tangible benefits to be a Champion. Mobile phones and the films served incentives, as did the receipt of a training certificate which they could add to their credentials for future work opportunities. Perhaps the most important factor was the sense of pride and enhanced self-image that helping one’s own community brought to the Champions (Fig. 1).

Fourth, films on the mobile phones facilitated dialogue and sharing of information on important mental health issues experienced in the community. Many Champions linked reductions in problems they observed specifically to people viewing these films. This could therefore be a helpful enabler of the *Atmiyata* intervention for future scale-up in other contexts (Fig. 1).

Fifth, close collaboration with local stakeholders enhanced acceptability and feasibility of delivering the intervention. The project’s local partner (a non-governmental organisation called BAIF) has been active in the intervention district for many years and has close contact and rapport with village members (including village-level decision-makers). This was important for the project’s initial credibility and obtaining buy-in from community members. BAIF helped identify suitable champions, assisted them with their work (e.g. to discuss and meet with complex cases, to help with managing time), and supervised their performance. In addition, having clear referral linkages in place with the nearest district hospital, and having Champions facilitate this referral and actively support accessing hospital care played an important role in improving access to care. Many community members (e.g. Mitras, families of Champions and individuals who had achieved functional recovery) played an important role in spreading awareness regarding *Atmiyata* and mental health, and in identifying and referring persons for care. It is therefore imperative that any scale up of *Atmiyata* should involve collaborations with local organisations who have strong links and networks within the community (Fig. 1).

At the population level, GHQ-scores were significantly lower among participants in the intervention district compared to the control district. We believe this could be due to the combination of factors discussed above (and summarised in figure Legend). It is important to note here that this was not designed as an efficacy trial, but rather as a pilot study to inform a larger planned implementation of the intervention.

### Limitations

There are several limitations to this study. First, due to logistical challenges and limited research capacity at the end of this project, we were not able to carry out qualitative interviews among all desired participant groups, namely intervention recipients. This information would have been valuable in obtaining a sense of the experience of care and help with further adaptation of the intervention in that particular context. Second, it is important to note that this was not designed as an efficacy trial for lay health worker delivered interventions, but rather a pilot study of a larger planned implementation research intervention, which is currently being carried out in the State of Gujarat as part of a scale-up project. The results of this study will confirm if the intervention is indeed effective. Our aim was an attempt to bridge the implementation gap that exists between the development of evidence-based interventions and their implementation in routine practice settings, and direct our research aims instead in assessing the interventions’ feasibility and appropriateness prior to large scale-implementation, the latter of which demands a rigorous research evaluation design. Third, the baseline difference in the mean GHQ scores is large and we don’t have any clear explanation for the same. We matched the two blocks on socio-demographics such as population structure, social group structure, occupation, geographical topography. The only major difference between the two blocks is that Trymbak block is a nationally known religious site where major festivities (Kumbh mela) happen. We have no evidence to make any association between this fact and the mean GHQ scores. However, this finding speaks to the need to identify newer criteria for selection of control groups for mental health studies.

Lastly, there is possibility that the reduction in GHQ + 6 scores from 65% to 36% may be due to natural remission, but it is unlikely. The absence of a control group hampers us from drawing any firm conclusions.

## Conclusion

The Atmiyata model could serve as a financially viable and sustainable model for task-shifting mental health identification, treatment, and follow-up tasks in contexts with limited resources, particularly with a shortage of human resources for mental health. This study shows the acceptability and feasibility of implementing a community-based intervention delivered by lay community members in a resource-scarce setting. Research is needed to test effectiveness, scalability and sustainability of this model when implemented at a scale.

## Supplementary information

**Additional file 1.** Selection and training of Champions and Mitras. The document briefly describes processes of selection and training for Champions and Mitras who are the volunteers from each village and core to the Atmiyata programme.

**Additional file 2.** Overview of counseling sessions. Each Champion delivers 4 to 6 counseling sessions to person with the distress and common mental health issues using basic counseling techniques of active listening, behavioural activation and problem solving. The document briefly describes the steps covered in each session.

**Additional file 3.** Success stories. This document contains experts from Champion’s interview and mentions Champion’s work impact on beneficiaries with common and severe mental health issues.

## Data Availability

Data and materials will be shared upon request by Dr. Kaustubh Joag, Centre for Mental Health Law and Policy, Indian Law Society, Pune, India.

## Abbreviations

CF: Community Facilitator
CMD: Common Mental Disorder
GHQ-12: General Health Questionnaire- 12
LMIC: Low- and- Middle-Income Country
PHC: Primary Health Centre
SHG: Self-Help Group
SMD: Severe Mental Disorder
