# Supplementary material for: Feasibility and acceptability of a novel community-based mental health intervention delivered by community volunteers in Maharashtra, India: the Atmiyata programme
Source: BMC Psychiatry. 2020 Feb 7;20:48. doi: 10.1186/s12888-020-2466-z (PMC7006077; doi:10.1186/s12888-020-2466-z)
Supplement: Supplementary file 1 — Additional file 1. Selection and training of Champions and Mitras. The document briefly describes processes of selection and training for Champions and Mitras who are the volunteers from each village and core to the Atmiyata programme. [file 12888_2020_2466_MOESM1_ESM.pdf]

Process of selection and training of Champions-

1. The selection criteria for community volunteers (Champion) has been developed. Champion should have a. willingness to work voluntarily; b. leadership qualities or a leader of Self-help group and Farmer's club; c. understanding of geography and social dynamics of the village community; d. readiness to give time for the training and actual work (1 hour per day for 6 days a week); e. basic literacy skills; f. interest and enthusiasm for the work.

2. Community Facilitator (CF) meets key people in the assigned village like village head, community leaders, health workers, and gets 5 to 6 names of potential volunteers. CF then visit these people separately and gives the detailed information about 'Atmiyata'. CF explain them their role and responsibility as a Champion. Once the person agreed then he or she is enrolled as 'Champion'.

3. 1 Champion is selected for 1000 to 1200 adult population in each village. Also, the caste and gender balance are maintained carefully while selecting Champions.

4. 7-days training spread over 3 weeks is arranged at central location. In one batch 25 to 30 Champions are trained. CF and project manager delivered the training.

Flow chart for selection and training of Champion (Period- 3 to 6 months)

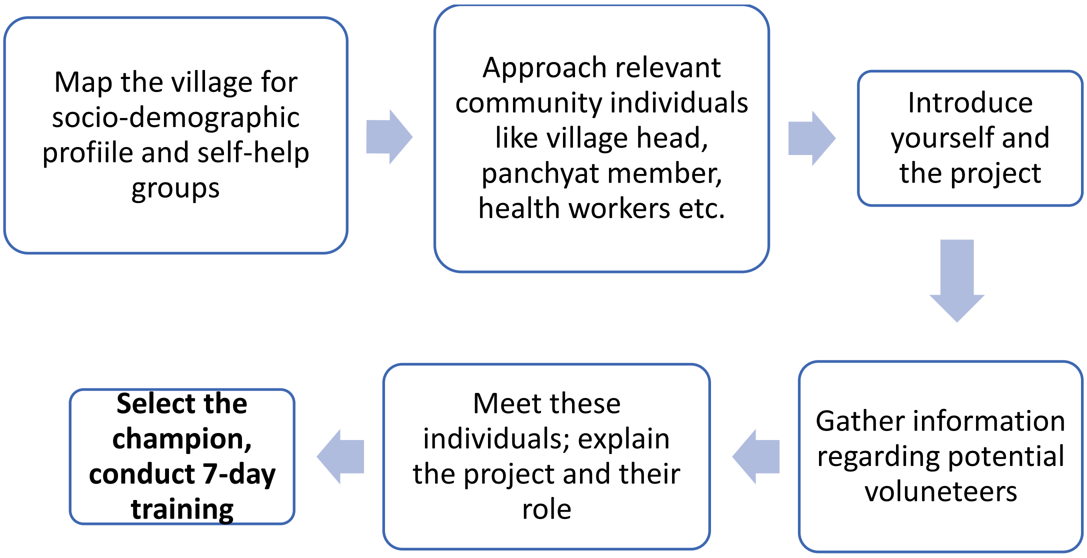

Process of Selection and Training of Mitra-

1. Champions select Mitras who are willing to work voluntarily and give time for training and work. Each Champion select 4 to 5 Mitras depending on geography and social barriers.

2. CF and Champion train Mitras which was 1-day training based on explaining Atmiyata, identification of CMD and SMD and role of Mitra. Also, Mitras are encouraged to show films or share films with others.
