# Supplementary material for: Feasibility and acceptability of a novel community-based mental health intervention delivered by community volunteers in Maharashtra, India: the Atmiyata programme
Source: BMC Psychiatry. 2020 Feb 7;20:48. doi: 10.1186/s12888-020-2466-z (PMC7006077; doi:10.1186/s12888-020-2466-z)
Supplement: Supplementary file 2 — Additional file 2. Overview of counseling sessions. Each Champion delivers 4 to 6 counseling sessions to person with the distress and common mental health issues using basic counseling techniques of active listening, behavioural activation and problem solving. The document briefly describes the steps covered in each session. [file 12888_2020_2466_MOESM2_ESM.pdf]

Champion's Counselling Sessions Overview

Champions take verbal consent before every session. Each session lasts for 20 to 40 mins.

Following table gives overview of steps which Champions followed during 4-6 counselling sessions.

Session 0	Session 1	Session 2	Session 3, 4 and 5	Session 6-
-Understand the distress, stressors and context of problems.<br>-Explain the nature, structure and purpose of counselling sessions.<br>-Plan for the sessions and decide time and place to meet.	- Conversation around getting to know client's goals<br><br>- Active listening skills (what is going on in your life/what are your problems?)<br><br>- Do they want a relative involved?<br><br>- Plan sessions ahead<br><br>-Active listening, empathy are the skills which are used in every session	-Problem solving techniques – how to solve problems?<br><br>-Introduce activity scheduling & activity log<br><br>-Make selection of activities to try before next session<br><br>-Schedule next appointment<br><br>- Relaxation exercise demonstration and encourage to practice	-Review homework and activity log from last session<br><br>-Ask how moods have changed due to activities<br><br>-Have discussion on mood changes/how activities made client feel<br><br>-Review problems<br><br>-Add new activities that align with goals stated from Session 1<br><br>-Relaxation exercise at the end of session if feasible	-Review goals<br><br>-Summarize skills learned<br><br>-Make a plan for how to respond to challenging situations<br><br>-Develop toolbox of helpful responses
