# Supplementary material for: Feasibility and acceptability of a novel community-based mental health intervention delivered by community volunteers in Maharashtra, India: the Atmiyata programme
Source: BMC Psychiatry. 2020 Feb 7;20:48. doi: 10.1186/s12888-020-2466-z (PMC7006077; doi:10.1186/s12888-020-2466-z)
Supplement: Supplementary file 3 — Additional file 3. Success stories. This document contains experts from Champion’s interview and mentions Champion’s work impact on beneficiaries with common and severe mental health issues. [file 12888_2020_2466_MOESM3_ESM.pdf]

Success stories

Target problems	Case description	Excerpt from the Interview
Alcohol use	A XYZ whose partner was drinking, started drinking as well and then stopped after counselling	Champ60224M11: (One person) XYZ's partner was an alcoholic. Due to tensions, she too started alcohol. I counselled XYZ regularly. XYZ had a lot of tensions. I talked to XYZ many times. Even made visits to XYZ. Now XYZ has left alcohol. XYZ' child is a nurse at nearby village. Now XYZ has saved some money.
	A person in the village who drank stopped drinking after counselling and seeing the films	Champ60224M04: There was one person – DRG. DRG had five children. DRG used to drink alcohol no matter what the cost was (to the family). Even if DRG had to steal and sell things from home (DRG would drink). Like even food grains (DRG would steal). DRG was creating such problems. Then I contacted DRG. I warned DRG, "If you steal, you will be arrested, you will be jailed and then you will understand life". I even took DRG to the police station. It has been a constant effort. Now DRG does not even mention alcohol (in a conversation). DRG is cured. DRG listened to me and took it to the heart and now the subject of drinking is stopped.
	A person in the village who drank stopped drinking after counselling	Champ60224M04: Another person… who is not yet married… the name is MDP. MDP was an alcoholic. I told MDP, "If you drink so much, no one will give marry you, you will die unmarried". MDP also did the same (left alcohol).
	A young person in the village who was drinking stopped after counselling	Champ60224M04: One person, SDP chose two partners for marriage. When a family is marrying their child to somebody, they will enquire about the partner, whether the partner drinks or (whether) anything else (is wrong with him). So, when they got to know that SDP drinks, SDP started drinking more. SDP was in tension. So, I went and told SDP, "If you are drinking because you are having tension then it does not reduce, it will increase instead. Keep this in mind". I told SDP everything. Now SDP has also reduced.
Domestic violence	A couple who were experiencing marital problems (in part, because of the partner's drinking) changed their respective behaviours after counselling	Champ60223M03: There is one family in the village. One adult ABC was addicted to alcohol and under its influence ABC used to beat partner. We talked to ABC, counselled ABC many times. Listened to ABC carefully, allowed ABC to open up (to us). ABC then shared many secrets with me. We even talked to ABC's partner. Counselled ABC's partner as well, told the partner to behave calmly with ABC, ABC will definitely change. ABC's partner was not behaving well. ABC's partner decided to change, and ABC also changed. ABC started loving her. We visited family five times. Now that family is living a healthy life.

Stress<br>Trauma<br>Depression	A person whose partner left after marriage experienced stress, but this reduced after counselling	Champ60224M03: One person… UB… my cousin's relative… UB got married but UB's partner went back own home the next day. After marriage the partner goes to parents' home the next day as a ritual. But UB's partner refused to come back. UB's partner said that - does not want to stay there (with UB). UB has a problem of stammering while talking. UB was in great tension. One day UB came to our farm. Then my family member asked UB "Why are you in tension?". UB said, "After marriage that too… after (me having) taken so much of money on a loan, my partner is saying that- will not come (home). What should we do then?". Another day, when UB came back again, I told UB, "Don't think so much. If you think so much then it is you who will be stressed. When people start thinking they think big (thoughts)". I told him, "Don't think too much, you have a family and take their support. Put your mind on your work.". We taught UB a little bit (what to do). But UB soon was better. "Let my partner go. I will marry again". Now UB says that will marry again.
	A person whose partner beat after drinking was worried and angry but after following the champion's advice improved.	Champ60224M03: In our village there was a person, ABC. ABC used to think a lot. One day we went to wash clothes, there is water nearby. ABC was very angry (naraaz). I asked ABC… "Why are you so silent?" ABC said, "Its nothing like that". But I insisted, "You have something in your mind. Tell me". ABC was not ready to tell me. Then ABC told me that her partner gave ABC a lot of trouble. "the partner drinks a lot. Then the partner beats me a lot". Then I asked ABC, "Have you spoken to your parents about this?". (She replied), "What should I tell them?!. They will get tension". I went to ABC's house. I gave ABC the mobile and asked ABC to see the film. I left the mobile with ABC. Then ABC saw, heard (the film) and told me that. Then I explained it to ABC and advised ABC to talk to her partner and tell "Do not trouble me, I will do all my work properly". After that day there is a change in ABC. She is behaving nicely.<br><br>Champ60224M03: Let me tell you a story from my own house. My relative XYZ was married… after a few days XYZ used to act like a "mental" because XYZ's partner left XYZ. After our marriage I was not allowed to go into XYZ's room. My relative told me that XYZ behaves in a very weird manner, one day XYZ was going to hang himself. That time I did not have any experience… no training. But during the training, when sir told us (about various illnesses). I immediately understood what it (the problem) was. Then, when I got the first film and the mobile, I asked my partner to accompany me to XYZ's room. I asked XYZ to listen to it. After seeing this XYZ said. "This is what happens because of a partner". XZ was earlier given treatments and also shocks. Even then, there has been no change in XYZ but now XYZ said, "I will not behave like this again".

		After that I also showed the film on Shobha and the others. Now XYZ is better. Then my partner also spoke to XYZ a lot and now XYZ is doing own farming. We showed XYZ the film, spoke to XYZ a lot. Told XYZ if you think a lot then you will have mental illness. At that time, XYZ used to sit alone and think. XYZ made efforts to become a little better.
	A person, ABD, who was having panic attacks after a traumatic experience felt better after ABD was referred to a PHC and got medication	Champ60224M03: Just about 15 days ago, there was a death in the family. My relative, ABD from Nashik had come home. ABD had not seen any death before. ABD got scared if the face of the dead body came in front of ABD. "My chest gets heavier", ABD used to hold own chest. I told ABD that you had taken that episode (death) to the heart (manat bibaun ghetlay). Then like Sir had told us, I told ABD about the breathing exercise. But it was not helping ABD. ABD would tell me to stop the topic (of relaxation) and put some songs on etc. ABD is yet to recover from it. I told ABD two, three times. ABD's partner took to the PHC. ABD feels better if ABD takes medicines. I tell ABD "When you take it think to yourself, 'taking these medicines will make me feel better'". ABD tells me that it is a little better now.
Suicidal attempts	A person who was drinking, and attempted suicide has reduced his drinking after seeing the films and receiving counselling<br><br>A person who was drinking, and attempted suicide has reduced his drinking after seeing the films, attending the champion's Satsang, and receiving counselling	Champ60223M05: One person- XYZ attempted suicide; I visited XYZ on a regular basis. When I came to know about that, I visited XYZ's place. Showed XYZ the films and counselled. XYZ was always drunk but has reduced now. XYZ talks in a good way with me. The family is okay now. They treat us well given that because of us XYZ is alive. They are very good people.<br><br>Champ60224M01: Both partners would constantly fight. The day ABC (one of the partners) used to drink, there would definitely be a fight. (One day) what ABC did during a fight… in a state of drunkenness was… ABC took poison. Then… our Manav Seva (self-help) Group… they helped me by removing the poison from ABC's body. Then when we brought ABC home, I asked ABC, "If you had died, what would your children have done after your death? Where would have the mother run for help? Being a parent, you earn some money and give food to your children so don't do this again". ABC does not know who God is. (ABC only knows) to drink alcohol, work whole day, (and) when money comes, to drink alcohol. But since ABC has come to our Satsang (religious activity) there is a lot of change in ABC's family. They are happy now. We showed ABC the Vyasnadin (addiction) Ganpat film. Now ABC is happy and has bought a vehicle for the child worth 65,000. ABC bought 2-3 buffaloes, started a milk selling business.
Severe mental illness	MDB who was previously ostracised by his village and his family was referred for	Champ60224M11: In my village there was one patient-MDB Villagers discriminated MDB calling him "Mad!''. Even today I go and give medicines to MDB. No one from MDB's family (used to) take care of MDB. MDB's partner and children

	psychiatric treatment, and is now being cared for by the child	discriminated against MDB, they left MDB long ago. Even today MDB is on medication. We took MDB to Civil (hospital) with Kaustubh sir. MDB is much better. Even today MDB spoke to me. No body used to talk to MDB. But MDB is very normal (now). MDB was given a diary for noting down the medicines (he takes). Doctor had told not to miss doses. The doctor also counselled MDB. MDB's child gives medicines. MDB's child takes care of MDB.
	A person (ABC) with a severe mental illness is now better after psychiatric treatment	Champ60224M04: It was a mental problem due to love failure. Now there are medicines… in the BAIF's vehicle we took ABC to Nashik civil (hospital). I also called ABC's sibling to go with us. Then they gave the medicines for 15 – 30 days. The doctor told ABC's sibling, "If ABC should take the medicines directly else give those in the food". And the sibling is doing it. Slowly, slowly ABC is getting better. ABC is at home only now. Not completely but yes there is change.<br><br>Champ60222M06: One patient who delivered (a baby) on the road. She was not in her senses. She did not do anything for the child. Did not feed, no cooking, nothing. It was a severe case. It was before our work. Her partner did magic related issues also to see if anything has happened to her. He took her to a private (doctor) but the treatment was not affordable for them. It stopped as the expenses were 2 to 3000 rupees. Then this (Atmiyata) program came. I met him. I told him about the program and its details. Her parents stayed with them. They were not agreeing (to take treatment). I talked to her husband over the phone as he was working in Nashik at that time and then talked to BAIF staff. A vehicle was organized. We took her to Civil (hospital). The doctor checked her. Her treatment is going on. She is recovering. She is doing everything now. Household work, daily wages, taking care of children.
